# Non-functioning parathyroid carcinoma: a case report

**DOI:** 10.1186/s40792-017-0357-4

**Published:** 2017-07-19

**Authors:** Nobuyasu Suganuma, Hiroyuki Iwasaki, Satoru Shimizu, Tatsuya Yoshida, Takashi Yamanaka, Izumi Kojima, Haruhiko Yamazaki, Soji Toda, Hirotaka Nakayama, Katsuhiko Masudo, Yasushi Rino, Kae Kawachi, Yohei Miyagi, Akio Miyake, Kenichi Ohashi, Munetaka Masuda

**Affiliations:** 1 Kanagawa Cancer Center, Yokohama, Japan; 2 Yokohama City University, Yokohama, Japan

**Keywords:** Non-functioning parathyroid carcinoma, RT-PCR

## Abstract

**Background:**

Non-functioning parathyroid carcinoma is a rare disease that is difficult to distinguish from other diseases based on the lack of hyperparathyroidism. This is a report of non-functioning parathyroid carcinoma diagnosed by reverse transcription polymerase chain reaction (RT-PCR) targeting parathyroid hormone (PTH) messenger RNA.

**Case Presentation:**

The patient is a 67-year-old male who visited our hospital for the chief complaint of hoarseness. A 5-cm mass was observed in the right lobe of the thyroid gland, and poorly differentiated thyroid carcinoma was suspected according to the fine-needle biopsy results. The laboratory data for thyroid functions, thyroglobulin, anti-thyroglobulin antibodies, calcium, phosphorus, and intact-PTH were all within the normal range. Right recurrent nerve paralysis was observed preoperatively. The patient was diagnosed with poorly differentiated thyroid carcinoma, and total thyroidectomy and central node dissection with partial resection of the right recurrent nerve and esophageal muscle were performed. The pathological findings revealed atypical cells containing clear cells in solid and alveolar structures with broad fibrosis. Mitosis, focal coagulative necrosis, and vascular and capsular invasions were observed. A slightly positive PTH immunohistochemical stain was noted, whereas the RT-PCR results were positive. We finally diagnosed this tumor as non-functioning PTC. No distant metastasis occurred, and the patient is still alive.

**Conclusions:**

This is a report of a patient with non-functioning parathyroid carcinoma, which is clinically very rare. We diagnosed this tumor as non-functioning parathyroid carcinoma using RT-PCR for PTH mRNA.

## Background

Parathyroid carcinoma (PTC) is a rare type of endocrine tumor that accounts for less than 5% of all cases of primary hyperparathyroidism. Most PTC patients have clinical manifestations of hyperparathyroidism. However, in some cases, patients may not present increased serum calcium or parathyroid hormone (PTH) levels. This type is called non-functioning parathyroid carcinoma, which is an extremely rare type reported in less than 30 cases. It is occasionally difficult to distinguish this disease from other diseases based on the lack of hyperparathyroidism. This is the first report of non-functioning parathyroid carcinoma diagnosed by reverse transcription polymerase chain reaction (RT-PCR) targeting PTH messenger RNA (mRNA).

## Case presentation

A 67-year-old male presented with hoarseness and was admitted to our hospital. Cervical examination revealed a 5-cm hard mass at the lower portion of right thyroid lobe fixed with the swallowing.

Laboratory findings demonstrated that all thyroid and parathyroid functions are within the normal range: thyrotropin (TSH) 3.68 μIU/m (normal range 0.50–5.00), free triiodothyronine (T3) 3.10 pg/ml (2.30–4.00), free thyroxine (T4) 1.06 ng/dl (0.90–1.70), thyroglobulin (Tg) 31 ng/ml (≤32.7), anti-thyroglobulin antibodies (TgAb) 10.0 IU/ml (<28.0), serum calcium 9.4 mEq/l (8.8–10.1), serum phosphorus 3.2 mg/dl (2.6–4.4), and intact parathyroid hormone (PTH) 49 pg/ml (15–65).

**Fig. 1:**
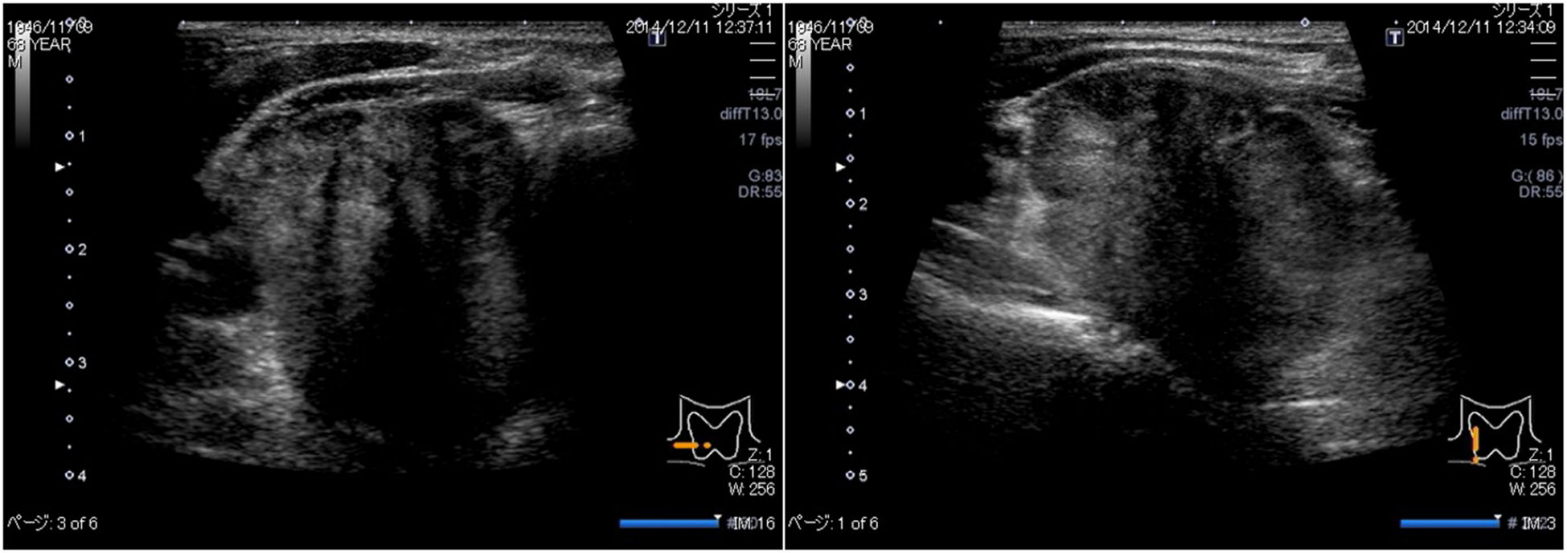
Ultrasound findings. A hypoechoic area of 4.7 × 3.4 cm with an irregular margin and heterogeneity was detected

**Fig. 2:**
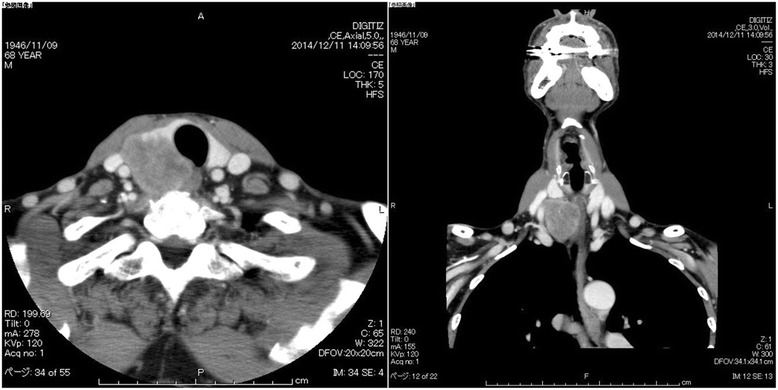
CT findings. A low-density mass of approximately 5.0 cm in the right lobe of the thyroid-induced tracheal and esophageal deviation and deformation

**Fig. 3:**
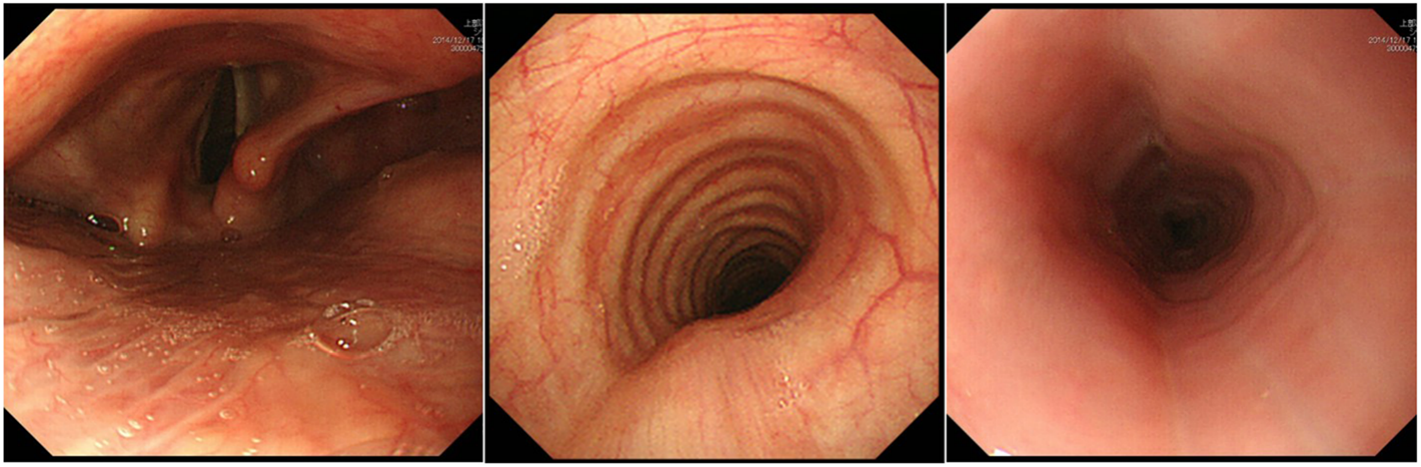
Endoscopic findings. Right vocal code paralysis and tracheal/esophageal deformation were detected through endoscope images. No abnormality was found in the tracheal mucosa

Ultrasonography (US) and computed tomography (CT) revealed a hypoechoic area of 4.7 × 3.4 cm with an irregular margin in the right lobe of the thyroid (Fig. 1). No lymphadenopathy was detected. CT findings revealed a low-density mass of approximately 5.0 cm in the right lobe of the thyroid. The tumor had close contact with the posterior side of the trachea and the right wall of the esophagus in broad areas. The margins are indistinct, and tumor invasion was suspected (Fig. 2). Neither cervical lymph node metastasis nor distal metastasis was observed. Endoscopic findings indicated that right vocal code paralysis was observed in bronchoscopy. The tracheal lumen was deformed due to retraction by the tumor from the right posterior side. No abnormalities were found on the tracheal mucosa. The upper endoscopy did not reveal any obstructions or abnormalities on the esophagus mucosa (Fig. 3).

Fine-needle aspiration revealed cells with a large N/C ratio. No cells exhibited nuclear grooves or intranuclear cytoplasmic inclusion, suggesting that it was a poorly differentiated thyroid carcinoma (PDTC) derived from a follicular tumor.

**Fig. 4:**
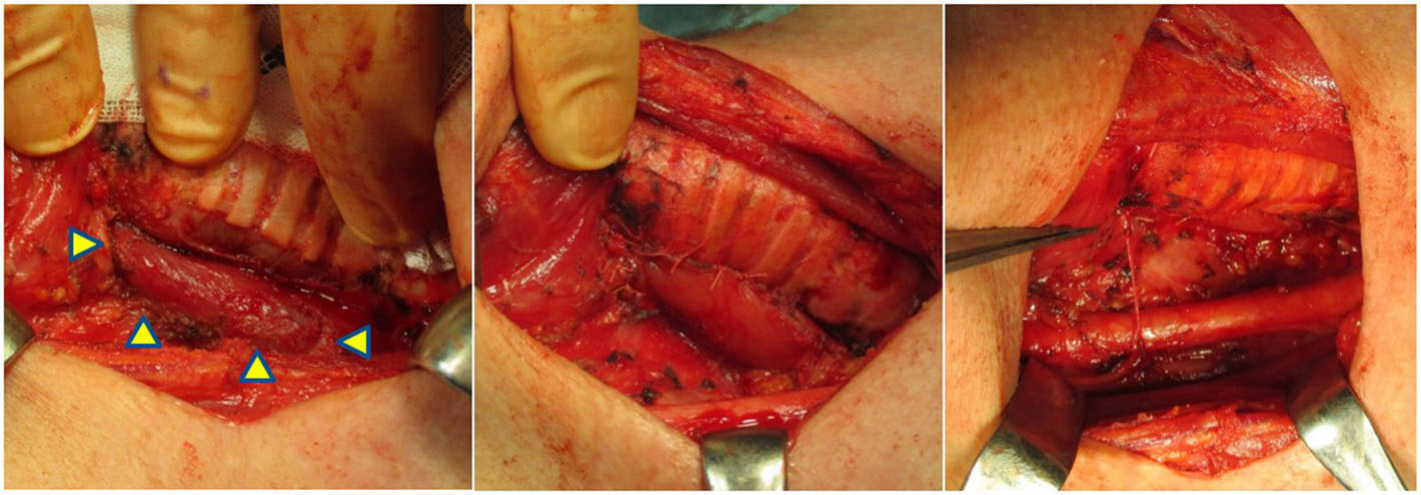
Surgical findings. The muscular layers of esophagus and trachea were resected and sutured. Nerve reconstruction was performed by end-to-end anastomosis between the right recurrent nerve and the right ansa cervicalis

A surgical treatment was performed, suspecting PDTC with right recurrent nerve, esophageal, and tracheal invasions. The tumor involved a right recurrent nerve and invaded the sternothyroid muscle on the ventral side and the muscular layer of esophagus and the membranous wall of trachea on the dorsal side. Some parts of the right recurrent nerve, the muscular layer of the esophagus, and the membranous wall of tracheal were resected with the tumor via thyroidectomy along with lymph nodes of the central compartment. The tumor was not macroscopically exposed. The defects of the muscular layer of the esophagus and the membranous wall of the trachea were sutured in the direction of the long axis. Then, nerve reconstruction was performed by end-to-end anastomosis between the right recurrent nerve and the right ansa cervicalis (Fig. 4).

**Fig. 5:**
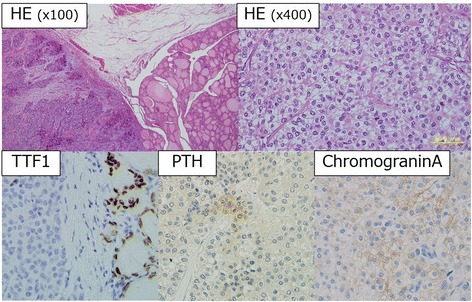
Pathological findings. A solid tumor with broad fibrosis invading surrounding tissues was observed under ×100 field, and atypical cells containing oval and clear cytoplasm were observed under ×400 field. The cytoplasm was slightly stained for PTH and chromogranin A. Negative TTF-1 staining was noted in cancer cells

The tumor was a solid gray white mass of 42 × 37 × 28 mm with an irregular surface invading the sternothyroid muscle, right recurrent nerve, the muscular layer of esophagus, and the membranous wall of trachea. Microscopic findings include a growing mass of atypical cells containing clear, partially oxyphillic cells, in solid and alveolar structures with broad fibrosis. Mitosis and focal coagulative necrosis were also detected. Vascular and capsular invasions were observed inside the tumor and the surrounding tissues; lymph node metastasis was positive (3/8). According to the immunostaining results, the tumor cells were slightly positive for PTH and chromogranin A and negative for TTF-1, thyroglobulin, calcitonin, CD5, and S100 proteins. The patient was diagnosed with parathyroid carcinoma, and the MIB1 index was 5% (Fig. 5).

**Fig. 6:**
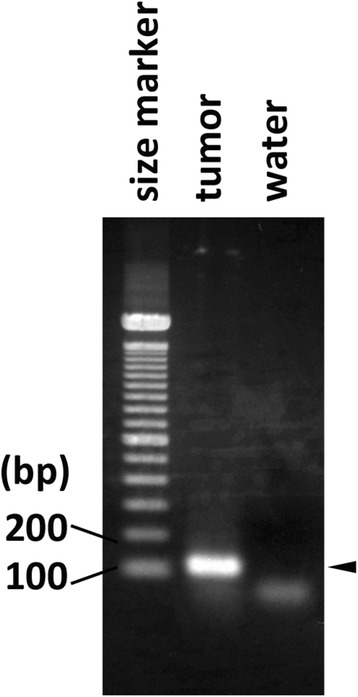
RT-PCR findings. Identification of the parathyroid hormone transcript in tumor RNA by RT-PCR. RNA extracted from the tumor and water was subjected to RT-PCR analysis for PTH transcript detection as described in the Materials and Methods section. The *arrowhead* on the *right side* indicates the band from the tumor sample that almost corresponds to the predicted PCR product size of 103 base pairs

PTH immunostaining was slightly positive and non-specific. Hence, PTH mRNA expression was analyzed by RT-PCR. Several thin-sliced sections were serially prepared on glass slides from the formalin-fixed paraffin-embedded (FFPE) tissues of the surgically removed tumor, and one of the slices was subjected to hematoxylin and eosin (H&E) staining. RNA was extracted from the area on the remaining unstained slides corresponding to the tumor identified on the H&E-stained slide using the AllPrep DNA/RAN Mini Kit (Qiagen, Germany) following the manufacturer’s protocol. The extracted tumor RNA was subjected to the RT-PCR analysis using the SuperScript® One-Step RT-PCR with Platinum® Taq kit (ThermoFisher Scientific, USA) according to the manufacturer’s protocol. Briefly, 1 μg of the extracted RNA or water as a negative control was reverse transcribed with the reverse PCR primer followed by 40 cycles of PCR amplification consisting of incubation at 94 °C for 15 s, 55 °C for 30 s and 68 °C for 30 s in one tube using the 2720 Thermal Cycler (ThermoFisher Scientific). The nucleotide sequences of the forward and reverse PCR primers were 5’-GCCAACATGACAATCATA-3’ and 5’-GTTTTCCCAGGTTATGCA-3’, respectively. These sequences were designed from exons 2 and 3 of the PTH transcript sequence deposited as NM_000315 in GenBank. RT-PCR products were detected by electrophoresis in a 2% agarose gel stained with ethidium bromide. PTH mRNA expression was observed, and finally we diagnosed this tumor as non-functioning PTC (Fig. 6).

The post-operative course was satisfactory, and no dysphagia was observed. The patient was discharged 5 days after the operation. Seven months later, a palpable lymph node of approximately 2 cm was detected on the right neck during a physical examination. The US and CT scans revealed lymphadenopathy in the right upper deep cervical lymph nodes. The patient was cytologically diagnosed with the parathyroid carcinoma. Then, right modified neck dissection was performed 9 months after the operation. Given that prophylactic irradiation therapy has a low evidence level and has not been established, we suggested the patient undergo radiation therapy, but he refused. Unfortunately, re-operation was needed for the right neck recurrent lymph nodes 25 months after the first surgery. We finally decided to apply radiation therapy to the neck after the third operation. No distal metastasis has occurred, and the patient is still alive.

## Discussion

Parathyroid carcinoma is a rare disease with a prevalence of 0.015 per 100,000 persons and is estimated to account for 0.005% of all cancers [1]. Most patients with parathyroid carcinoma exhibit hyperparathyroidism, i.e., 2-5% of all primary hyperparathyroidism cases [2–4]. The median age is 48 years old. The male to female ratio for parathyroid carcinoma is approximately 1:1, whereas that for primary hyperparathyroidism is 1:3.5. More females are affected than males [5]. Clinical findings of parathyroid carcinoma include neck tumor, high PTH and associated hypercalcemia, and generalized fibrous osteitis [6, 7]. In addition, a report also states that findings such as D/W ratio ≥1 in the ultrasound scan and a tumor growing into the thyroid gland could be useful findings indicating malignancy [8]. Cytology is contraindicated as it may induce dissemination. Hence, it is necessary to develop treatment strategies against cancer when parathyroid carcinoma is suspected based on the above mentioned clinical findings. The gene involved in the onset of parathyroid carcinoma is hyperparathyroidism 2 (HRPT2), which is a tumor suppressor gene on a long arm of the chromosome 1 (1q31). Genetic mutations of this gene inactivate parafibromin. This gene has been identified as a causal factor of hyperparathyroidism-jaw tumor syndrome, a hereditary hyperparathyroidism condition, in 2002 [9]. Mutations in this gene have also been detected in sporadic parathyroid carcinoma [10].

In addition, non-functioning parathyroid carcinoma is a very rare disease, with approximately less than 30 cases reported to date [5, 11]. Genetic mutations in HRPT2 have also been reported with this disease [12]. However, given that insufficient data are available, further accumulation of cases is awaited. Unlike functioning parathyroid carcinoma, this condition does not develop hypercalcemia and associated clinical symptoms. Hence, non-functioning parathyroid carcinoma is typically discovered with neck tumor, hoarseness, dysphagia, or dyspnea [13]. Our case was also discovered with hoarseness without hypercalcemia or high PTH value; it was difficult to make a diagnosis preoperatively. A histopathological study revealed fibrous band formation inside the tumor, trabecular alignment of tumor cells, mitotic figures, and capsule/blood vessel invasions as described in the criteria proposed by Schantz and Castleman [14] and compatible with those of parathyroid carcinoma. In addition, we also performed immunostaining methods to differentiate this case from other diseases, such as PDTC, medullary thyroid carcinoma (MTC), carcinoma exhibiting thymus-like differentiation of the thyroid (CASTLE), paraganglioma, and thymic carcinoid. According to the results, staining for PDTC, TTF-1 and Tg was negative, and no differentiated cancer tissues were detected. For MTC, calcitonin was negative. For CASTLE, CD5 was negative. For paraganglioma, S-100 protein was negative. Hence, these diseases were excluded. For thymic carcinoid, it was difficult to differentiate using the immunostaining technique as both are neuroendocrine tumors. Although the tumor cells were slightly positive for PTH, the diagnosis of non-functioning parathyroid carcinoma was finally made by detecting PTH by RT-PCR, which offers increased sensitivity compared with immunohistochemical staining.

For the treatment of non-functioning parathyroid carcinoma, it is crucial to perform radical excision in the initial operation. It is difficult however to achieve a definitive diagnosis of this disease pre-operatively unless we keep this disease in mind, as it exhibits neither hypercalcemia nor PTH elevation. Radical excision should not be performed in the initial operation in approximately 86% of cases [1]. The lesions on the right recurrent nerve, muscular layer of the esophagus, and the membranous wall of trachea were all resected with the tumor during the initial operation. In our case, lymph node recurrence in the right neck occurred after 9 and 25 months from the initial operation, and irradiation to the neck was required after the second lymph node dissections. No distal metastasis occurred, and the patient is still alive.

## Conclusions

This is a report of a patient with non-functioning parathyroid carcinoma, which is clinically very rare. We diagnosed this tumor as non-functioning PTC using RT-PCR for PTH mRNA.

## Abbreviations

CASTLE: Carcinoma showing thymus-like differentiation of the thyroid
CT: Computed tomography
FFPE: Formalin-fixed paraffin-embedded
H&E: Hematoxylin and eosin
HRPT2: Hyperparathyroidism 2
MTC: Medullary thyroid carcinoma
PDTC: Poorly-differentiated thyroid carcinoma
PTC: Parathyroid carcinoma
RT-PCR: Reverse transcription polymerase chain reaction
T3: Triiodothyronine
T4: Thyroxine
Tg: Thyroglobulin
TgAb: Anti-thyroglobulin antibodies
TSH: Thyrotropin
US: Ultrasonography

## References

[1] Hundahl SA, Fleming ID, Fremgen AM (1999). Two hundred eighty-six cases of parathyroid carcinoma treated in the U.S. between 1985-1995: A National Cancer Data Base Report. The American College of Surgeons Commission on Cancer and the American Cancer Society. Cancer.

[2] Shane E, Bilezikian JP (1982). Parathyroid carcinoma: a review of 62 patients. Endocr Rev.

[3] Obara T, Fujimoto Y (1991). Diagnosis and treatment of patients with parathyroid carcinoma: an update and review. World J Surg.

[4] Sharretts JM, Kebebew E, Simonds WF (2010). Parathyroid cancer. Semin Oncol.

[5] Shane E (2001). Clinical review 122: parathyroid carcinoma. J Clin Endocrinol Metab.

[6] Levin KE, Galante M, Clark OH (1987). Parathyroid carcinoma versus parathyroid adenoma in patients with profound hypercalcemia. Surgery.

[7] Iihara M, Suzuki R, Kawamata A (2012). Onset mechanism of parathyroid carcinoma and its diagnosis and treatment. J Jpn Assoc Endocr Surg Japanese Soc Thyroid Surg.

[8] Hara H, Igarashi A, Yano Y (2001). Ultrasonographic features of parathyroid carcinoma. Endocrine J.

[9] Carpten JD, Robbins CM, Villablanca A (2002). HRPT2, encoding parafibromin, is mutated in hyperparathyroidism-jaw tumor syndrome. Nat Genet.

[10] Shattuck TM, Valimaki S, Obara T (2003). Somatic and germ-line mutations of the HRPT2 gene in sporadic parathyroid carcinoma. N Engl J Med.

[11] Wilkins BJ, Lewis JS (2009). Non-functional parathyroid carcinoma: a review of the literature and report of a case requiring extensive surgery. Head Neck Pathol.

[12] Guarnieri V, Scillitani A, Muscarella LA (2006). Diagnosis of parathyroid tumors in familial isolated hyperparathyroidism with HRPT2 mutation: implications for cancer surveillance. J Clin Endocrinol Metab.

[13] Giessler GA, Beech DJ (2001). Nonfunctional parathyroid carcinoma. J Natl Med Assoc.

[14] Schantz A, Castleman B (1971). Parathyroid caicinoma. A study of 70 cases. Cancer.

